# Tempo and mode of early gene loss in endosymbiotic bacteria from insects

**DOI:** 10.1186/1471-2148-6-56

**Published:** 2006-07-18

**Authors:** F Delmotte, C Rispe, J Schaber, FJ Silva, A Moya

**Affiliations:** 1 UMR Santé Végétale (INRA-ENITAB), INRA BP81, 33883 Villenave d'Ornon Cedex, France; 2 UMR Biologie des Organismes et des Populations appliquée à la Protection des Plantes [BIO3P], INRA BP 35327, 35653 Le Rheu Cedex, France; 3 Max Planck Institute for Molecular Genetics, Ihnestrasse 63–73, 14196 Berlin, Germany; 4 Instituto Cavanilles de Biodiversidad y Biologia Evolutiva, Universidad de Valencia, A.C. 22085, 46071 Valencia, Spain

## Abstract

**Background:**

Understanding evolutionary processes that drive genome reduction requires determining the tempo (rate) and the mode (size and types of deletions) of gene losses. In this study, we analysed five endosymbiotic genome sequences of the gamma-proteobacteria (three different *Buchnera aphidicola *strains, *Wigglesworthia glossinidia*, *Blochmannia floridanus*) to test if gene loss could be driven by the selective importance of genes. We used a parsimony method to reconstruct a minimal ancestral genome of insect endosymbionts and quantified gene loss along the branches of the phylogenetic tree. To evaluate the selective or functional importance of genes, we used a parameter that measures the level of adaptive codon bias in *E. coli *(i.e. codon adaptive index, or CAI), and also estimates of evolutionary rates (Ka) between pairs of orthologs either in free-living bacteria or in pairs of symbionts.

**Results:**

Our results demonstrate that genes lost in the early stages of symbiosis were on average less selectively constrained than genes conserved in any of the extant symbiotic strains studied. These results also extend to more recent events of gene losses (i.e. among *Buchnera *strains) that still tend to concentrate on genes with low adaptive bias in *E. coli *and high evolutionary rates both in free-living and in symbiotic lineages. In addition, we analyzed the physical organization of gene losses for early steps of symbiosis acquisition under the hypothesis of a common origin of different symbioses. In contrast with previous findings we show that gene losses mostly occurred through loss of rather small blocks and mostly in syntenic regions between at least one of the symbionts and present-day *E. coli*.

**Conclusion:**

At both ancient and recent stages of symbiosis evolution, gene loss was at least partially influenced by selection, highly conserved genes being retained more readily than lowly conserved genes: although losses might result from drift due to the bottlenecking of endosymbiontic populations, we demonstrated that purifying selection also acted by retaining genes of greater selective importance.

## Background

The smallest genomes belong to bacterial obligate pathogens or intracellular symbionts of eukaryotes (e.g., *Mollicutes*, *Rickettsiae*, *Spirochetes, Chlamydiae*, and insect-associated gamma-proteobacteria). Phylogenetic analyses indicate that their small genome size is a derived state accompanying the transition to their specialized intracellular lifestyle. In other words, all these microbes certainly evolved from ancestors with larger genomes. One of the smallest genome described to date belongs to *B. aphidicola*, gamma-proteobacteria that maintain mutualistic endosymbiotic associations with aphids [1]. Gamma-proteobacteria include other obligate partners of insects and command special attention for several reasons: first, genome reduction in this group has been extreme, yielding a ten fold range of genome sizes; second, free-living relatives of these symbionts include the model organism *Escherichia coli *(*E. coli*) for which extensive genetic information is available; third, gene order is extremely well conserved between symbionts of the same genus [2,3] and rather well conserved over genome fragments between symbionts and free-living relatives [4]. This allows the reconstruction of gene loss events at different evolutionary steps of symbiosis. The first genome sequence of *B. aphidicola *BAp [5] initiated comparative studies shedding light on the process of genome reduction in this endosymbiont [6,7] but the recent sequencing of two additional *B. aphidicola *strains [2,8] and of three endosymbionts closely related to *Buchnera*, i.e. *Blochmannia floridanus *and *Blochmannia pennsylvanicus *(the endosymbionts of carpenter ants, respectively *Camponotus floridanus *and *Camponotus pennsylvanicus*; [3,9]), and *Wigglesworthia glossinidia *(the endosymbiont of the tsétsé fly, *Glossina brevipalpis*; [10]) considerably increase the scope for comparative analysis to reveal evolutionary patterns of gene loss. Moreover, it provides the opportunity for assessing the generality of the process of genome size reduction in three kinds of symbiotic lineages that have different gene content shaped by specific nutritional needs of their insect hosts.

The identification of long term evolutionary forces that drive genome shrinkage in endosymbionts is much debated. The trend toward large scale gene loss could reflect the inefficiency of natural selection at maintaining genes in the genomes of these cytoplasmically inherited bacteria. Indeed, the vertical partitioning of symbiotic lineages among hosts and their reduced population sizes strongly favour drift, a mechanism proposed to be responsible for the accumulation of mildly deleterious mutations in symbiotic genomes [6,11]. The hypothesis of an increased fixation of deleterious mutations is supported by several lines of evidence: the general acceleration of evolutionary rates and massive AT enrichment [[12,13]; but [14] for a selectionist interpretation], the increased proportion of non-synonymous mutations [12], the loss of adaptive codon bias [15] and the very low level of intraspecific polymorphism observed in bacterial populations [16,17]. Because the host environment provides metabolites, many bacterial loci would become redundant thereby accumulating slightly deleterious mutations by a process known as Muller's ratchet, which eventually could lead to the functional inactivation of non essential genes. In fact, the DNA of a pseudogene may be completely deleted from the *B. aphidicola *genome in 40 to 60 My [18]. Even some apparently beneficial genes (DNA repair, transcriptional regulation and replication initiation mechanisms) have been lost confirming that drift may play an important role in genome reduction [5,9,19].

If the ultimate forces leading to genome shrinkage in endosymbionts are still much debated, the proximal mechanisms responsible for DNA removal seem to be better understood [20,21]. In particular, it has been suggested that DNA removal occurs because of mutational bias favouring deletions over insertions in bacterial genomes [22]. This process which is apparently universal in prokaryotes could account for the scarcity of non coding DNA in most bacterial lineages. The loss of genes involved in DNA repair which are otherwise broadly conserved in bacteria might explain why deletion biases are not "corrected" and eventually lead to gene degradation and loss. Strikingly, such losses are a common characteristic of *Buchnera*, *Wigglesworthia *and *Blochmannia*, but also of *Sitophilus orizae *primary endosymbiont (a younger symbiont with a larger genome, where two of such genes are inactivated [23]) and even of free-living marine bacteria recently engaged in genome reduction [24].  Chromosomal rearrangement which can lead to large deletion events as also contributes to DNA removal. Finally, obligate bacterial mutualists have lost the ability to incorporate foreign DNA which grants them a "one-way ticket to genome shrinkage".

Understanding the evolutionary processes that drive genome reduction requires determining the tempo (rate) and the mode (size and types of deletions) of gene losses. Detailed information is available on recent events of gene loss from comparing closely related symbionts: for example extreme genome stasis (conservation of gene order and gene repertoires) has been shown for three *B. aphidicola *genomes [2,8] with an estimated tempo of gene loss in the range of 2–3 Myr per gene. Similar patterns emerge for *Blochmannia *[3], where a comparison between two species revealed a limited number of gene losses scattered along the genome with different rates for the two species (*ca *0.6 Myr per gene for *B. floridanus *and *ca *6 Myr per gene for *B. penssylvanicus*). These findings of gradual gene loss with some degree of specific variation is compatible with a phenomenon of drift, as seen above, mitigated by selective processes, since gene loss affects differential gene functions for different symbionts (e.g. *B. aphidicola strains *tend to conserve many genes involved in amino acid synthesis, while *Blochmannia *species tend to conserve a disproportionate number of genes involved in synthesis of cofactors).

In contrast, less is known about the rhythm and types of gene loss that occurred in the early steps of the acquisition of symbiosis. This is particularly critical, because gene losses that occurred in the ancestor of all *B. aphidicola *strains or *Blochmannia *species respectively were massive (estimated to >1000 genes lost [25]). Two recent comparative studies which reconstructed the hypothetical ancestral genome of *B. aphidicola *BAp and its free-living relatives have reached diverging conclusions on the organisation of gene losses. Moran and Mira [6] found that early gene loss involved deletions of large sets of contiguous genes including loci with unrelated functions, possibly due to recombination at repeated sequences [2,26]. Such massive and non-oriented process would be the sign of strong genetic drift and weakened selection in the early stages of endosymbiosis [6,11,27,28]. In contrast, Silva *et al*. [25] who focused on blocks of conserved synteny, found that genome shrinkage arose through multiple events of gene disintegration dispersed over the whole genome, which would be better explained by selection acting on individual genes even in these early steps. Even if larger deletions could not be ruled out, these authors insisted on the importance of selection for explaining the genome shrinkage observed in endosymbionts. Interestingly, recent genomic and experimental data support both scenarios: on the one hand, the presence of hundreds of pseudogenes scattered around the genomes of some pathogens recently sequenced [29-32] support the scenario whereby genome reduction occurs by slow erosion of individual genes. On the other hand, Nilsson *et al*. [33] have nicely showed that, even on a short evolutionary time scale, the disappearance of large stretches of DNA can be frequent in bacteria establishing in a constant environment. This experimental result suggests that large-scale deletions may occurred during the initial stages of genome reduction [33].

Characterizing the final set of genes of reduced genomes should help resolve this debate. Genome shrinkage is largely a lineage-specific process [34]. Indeed, because evolution is a highly contingent process, it is difficult to predict the fate of each single lineage. Moreover, successive losses are not independent events because the loss of a gene from a genome may influence the types of losses tolerated in the future. Finally, identical functions can be achieved by non-orthologous genes in different lineages. For example, the comparison of genomes of closely related insect endosymbionts shows that they share only 50% of their protein-coding genes. Although important functions such as cell division processes, information storage and processing show a high conservation of their gene repertoires, remarkable differences exist for those genes that encode proteins involved in the cell envelope, flagellum biosynthesis and the metabolism of amino acids, nucleotides, or coenzymes [9,34]. However, this does not necessarily mean that gene loss is a completely random process. In eukaryotes, Krylov *et al*. [35] recently investigated the relation between the propensity of a gene to be lost and its functional importance. Interestingly, these authors found significant relationships between the "propensity for gene loss" (PGL) and sequence substitution rate, gene dispensability, the number of protein interactions and the expression level of the gene. Thus, at least in eukaryotes, the likelihood of being lost seems to be an inverse relation of the biological importance of a gene. To date, this hypothesis has not been tested in prokaryotes, possibly because it is difficult to disentangle the respective contribution of gene loss and horizontal gene transfers in the evolution of bacterial genome size [36]. However, we propose that the well characterized group of insect endosymbionts provides a good model system for investigating the relative importance of drift and purifying selection in the process of genome shrinkage.

In this study, we used five available endosymbiotic genome sequences of the gamma-proteobacteria – *B. aphidicola *BAp, *B. aphidicola *BSg, *B. aphidicola *BBp, *Wigglesworthia glossinidia *(Wgl) and *Blochmannia floridanus *(Bfl) – to test whether gene loss was related to the selective importance of genes. We used a parsimony method to reconstruct a minimal ancestral genome of insect endosymbionts and quantified gene loss along the branches of the phylogenetic tree. This allowed us to assess different loss events at different evolutionary scales and to estimate the likelihood that a gene be lost during the evolution of symbiosis. To evaluate the selective or functional importance of genes, we used a parameter that measures the level of adaptive codon bias in *E. coli *(i.e. codon adaptive index, or CAI), and also estimates of evolutionary rates (Ka) between either pairs of orthologs in free-living bacteria or in pairs of symbionts. These parameters are expected to be correlated with the selective pressure on the genes. We also studied the distribution of deletion sizes (in number of loci) in order to determine whether they occurred by small steps or through large deletions of multiple loci at different positions on the phylogenetic tree rooted at the last common ancestor of free living bacteria and modern endosymbionts.

## Results and discussion

### Single or multiple origin(s) of endosymbiosis?

**Figure 1:**
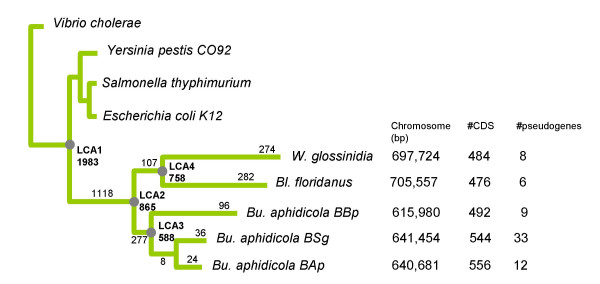
Phylogenetic tree based on an alignment of 61 concatenated conserved protein-coding genes involved in translation. DNA sequences were aligned using a protein alignment from clustalw, and then trimmed with Gblocks and limited to the first two codon positions resulting in 19143 nucleotides. The tree was reconstructed with NHML version 3 (Galtier 1998). *LCA *= Last Common Ancestor; Numbers in bold at the nodes indicate the number of protein coding genes (CDS) present at these steps. Numbers below the branches indicates the number of lost CDS since the last node. In front of each endosymbiotic lineage were indicated the chromosome size, the number of CDS and that of pseudogenes (assimilated to gene losses).

To place each gene loss event in an evolutionary context, we needed a reliable species tree onto which gene losses could be mapped. Our most parsimonious phylogenetic reconstruction yielded a monophyletic group containing the five symbiont lineages (Figure 1). The alternative tree topologies, in particular the scenario supporting a non-sister origin of these endosymbionts, showed significantly lower likelihoods, which confirm that the symbiosis between these five endosymbionts and their hosts resulted from an infection from a single bacterial strain. This does not mean that a single ancestral infection occurred that would have been followed by cospeciation of hosts and their symbionts. Rather, we may assume that a same species of bacteria tended to cohabit with species of insects belonging to different orders, and managed several times to establish symbiosis.

It is worth noting that the same result was recently found in at least three other studies, applying different methods on a different set of orthologous protein-coding genes [9,37,38]. In particular, Lerat *et al*. [38] insisted on the complete lack of conflict between 205 orthologous genes they chose resulting in a fully resolved phylogeny where *B. aphidicola *BAp and *Wiggleworthia *grouped together. In this context, we believe that the monophyletic topology of these five endosymbionts is a good "working hypothesis" although our phylogenomic approach precludes classical bootstrapping testing of the robustness of the tree.

However, Herbeck *et al*. [39] using a similar approach with more taxa but only two genes, the conserved groEL and 16S rRNA, proposed a different scenario. They found various phylogenies that group *Blochmannia *and *Wigglesworthia *but separately from *Buchnera*, which leaded them to conclude that *Blochmannia *and *Wigglesworthia *represent an origin of primary endosymbiosis that is independent from that of *Buchnera*.  Another recent study also cast in doubt the monophyletic character of the three endosymbiotic lineages [4]. These authors found a strong discordance between a phylogeny based on concatenated conserved amino acid sequences and reconstructions based on gene order. The former supported a single origin, while the latter placed *Blochmannia *as closer relative to *E. coli *than *Yersinia *and any other symbiotic lineage. This comes from the fact that synteny is high between *Blochmannia *and *E. coli *[3]. This result could still be compatible with a single origin for endosymbionts, provided that different levels of gene rearrangements occur among different lineages.

Although we cannot definitively conclude on the single/multiple origin of AT-rich endosymbionts, we have retained the monophyly of endosymbionts as a working hypothesis since it has been reached several times by different phylogenomic approaches, including our.

### Gene loss and CAI of *E. coli*

**Figure 2:**
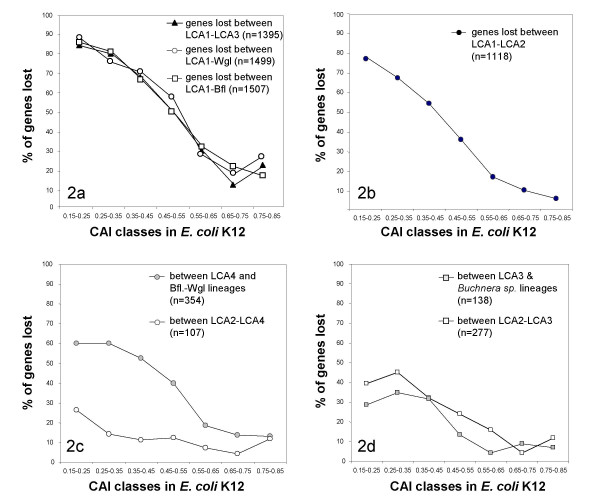
Relationship between frequency of gene loss in endosymbionts and adaptive codon bias (CAI) of the *E. coli *ortholog at different depths of the tree. y-axis: percentages of genes lost at least on one occasion between different nodes (or a node and a tip), x-axis: CAI classes of *E. coli *K12.

At all evolutionary scales low CAI genes were more readily lost than high CAI genes (Figure 2). Significantly (U-test, P < 0.001), gene loss was particularly biased towards low CAI genes early in the acquisition of symbiosis, since more than 80% of the sequences with CAI <0.25 (in *E. coli*) were lost between LCA1 (last common ancestor of endosymbionts and their free-living relatives) and LCA2 (last common ancestor of endosymbionts), representing 123 genes out of 158, while fewer than 20% of sequences with CAI >0.55 were lost, representing 22 genes out of 166. This result is not dependent on the hypothesis of common origin of all endosymbionts. Indeed, the pattern of loss is similar between LCA1 and *Blochmannia*, LCA1 and *Wigglesworthia*, LCA1 and the common ancestor of *B. aphidicola *strains (LCA3) or LCA1 and LCA2 (Figures 2a, 2b).

The proportion of genes lost in each CAI class within the symbiont clade did not exceed 60% (Figure 2c), probably because most low CAI genes had already been lost. However, even within the symbionts, three times as many low CAI than high CAI genes were lost (Figures 2c, 2d). Consequently, we find a significant increase in the mean CAI of lost genes along the tree which is also illustrated by the negative correlation between the propensity of a gene (PGL) and the CAI (Table 1).

**Table 1:** Correlations coefficients (R) between Propensity of Gene Loss (PGL) and Codon Adaptive Index (CAI) or non-synonymous substitution rates (Ka). The analysis is restricted to the genes present in the LCA2 that have been lost at least one time during symbiosis (Figure 3). All coefficients are highly significant (non parametric correlation test of Spearman; *P *< 0.005). N: number of genes.

	**N**	**R**
**CAI**	*1983*	-0.435
**Ka**		
*Eco-Ype*	*1363*	0.355
*Eco-Stm*	*1949*	0.248
*Wgl-Bfl*	*262*	0.244
*BAp-BSg*	*516*	0.147
*BAp-BBp*	*286*	0.178
*BSg-BBp*	*308*	0.209

### Gene loss and substitution rates

**Figure 3:**
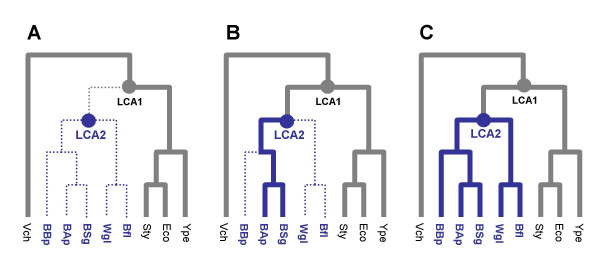
Three scenarios for ancestral genes: (A) Gene lost in all endosymbionts, presumably between LCA1 and LCA2 (B) Gene present in LCA2 but lost in some of the symbiotic lineages – here is a particular example were a gene has been lost independently in BBp, Wgl and Bfl (C) Gene kept in all endosymbionts. Solid lines represent the presence and dashed lines the absence of a gene.

**Figure 4:**
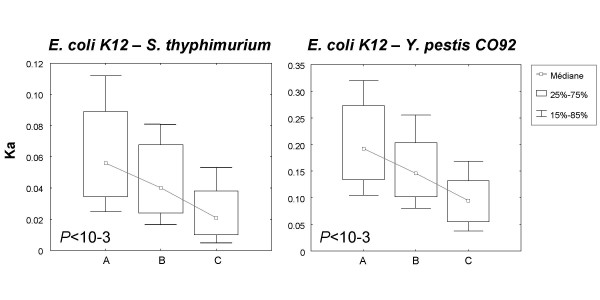
Non-synonymous substitution rates calculated between *E. coli *and two free living bacteria (*S. typhimurium *and *Y. pestis*) for each loss scenario (A, B, C correspond to gene loss in all, some or none of the symbionts respectively). Global significance of differences between medians was tested by Mann-Whitney tests.

The pairwise estimates of non-synonymous substitution rates between two free-living species pairs (Eco-Stm, Eco-Ype) were averaged for three different categories of genes: (A) Genes lost during the transition to symbiosis, i.e. between LCA1 and LCA2; (B) Genes present in the common ancestor of all symbionts but lost in some symbiotic lineages; (C) Genes retained in all endosymbionts (Figures 3, 4). We found a significant (U-test, P < 0.01) decline of Ka from (A) to (C) indicating that genes lost later were more conserved, hence probably of greater selective importance. This result demonstrates that the selective importance of a gene in free-living bacteria predicts its propensity of being lost in endosymbionts.

**Figure 5:**
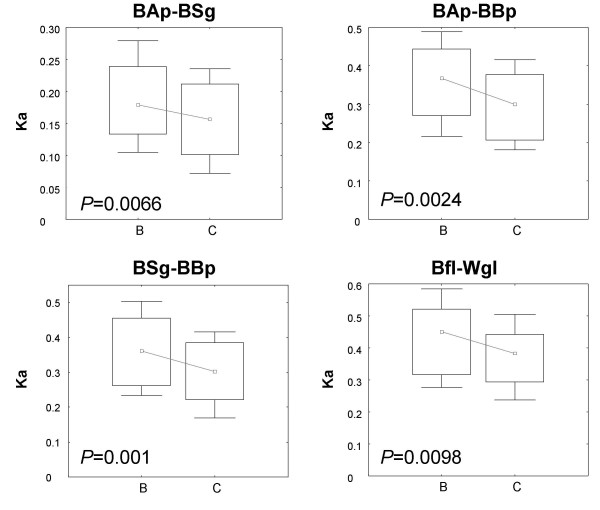
Non-synonymous substitution rates (Ka) calculated between pairs of endosymbionts for two different gene loss scenarios across all endosymbionts (B) Genes lost at least once, in some other symbiotic lineage; (C) Genes kept in all endosymbionts.

We also examined the non-synonymous substitution rates estimated for four endosymbiotic species pairs (BAp-BSg, BAp-BBp, BSg-BBp, Bfl-Wgl): for all comparisons tested, we found a similar evolutionary signal within the endosymbiont clade. Genes lost at least once within endosymbionts (B) were significantly less conserved than genes never lost (C), suggesting once again that those genes that were lost were of less essential function (Figure 5).

In consequence, we found significant positive correlations between PGL and the non-synonymous substitution rate for the five pairwise comparisons conducted (Table 1). Genes lost over the course of symbiosis evolution were faster evolving than average (in symbiont and free living bacteria).

Interestingly, CAI was negatively correlated with non-synonymous substitution rates both in symbiotic bacteria (four different comparisons tested) and free-living bacteria (two comparisons tested, Table 2). It is noteworthy that greater conservation of high expression genes at non synonymous site has been recently described in endosymbionts [40]. These results and ours confirms, also for prokaryotes, previous findings that highly expressed genes appear to evolve slowly [35,41].

**Table 2:** Correlation (R) between CAI and Ka for several pairwise comparisons between free-living and endosymbiotic bacterial lineages. All coefficients are highly significant (non parametric correlation test of Spearman; *P *< 0.005).

**Pairwise comparisons**	**R**
*Eco-Ype*	-0.491
*Eco-Stm*	-0.447
*Bfl-Wgl*	-0.374
*BAp-BSg*	-0.361
*BAp-BBp*	-0.387
*BSg-BBp*	-0.400

### Why some genes are retained and some are lost?

We have tried to determine the extent to which gene loss is predictable, by evaluating the correlations between some evolutionary parameters and the propensity of a gene to be lost. We showed that losses are concentrated on the genes that are evolutionary less constrained and of probably less selective importance (genes characterized by low adaptive codon bias in *E. coli *and high evolutionary rates in free-living and symbiotic species). The bias between propensities of loss for different categories of genes (genes of different CAI in *E. coli *and of different non-synonymous substitution rates) is particularly strong at the initial steps of the acquisition of symbiosis. We indeed demonstrated that at all stages of symbiosis, genome reduction was specifically targeted to genes of lesser selective importance, or less essential for the survival of the host/symbiont couple. It also is significant that symbionts retain some informative signal about the propensity of gene loss across subsequent diversification in their hosts: indeed, gene losses in later stages concern primarily sequences characterized by relatively high evolutionary rates in other symbiotic lineages. Late gene losses also primarily affect genes characterized by low CAI in *E. coli*. All this suggests that gene loss, even though governed by drift, was also partially influenced by selection both at ancient and recent stages of symbiosis evolution.

There is no a priori reason why non-synonymous substitution rate should be correlated with the propensity of a gene to be lost. Indeed, these two variables are measures of evolutionary conservation that capture substantially different aspects of evolution and one can imagine that functional constraints could be relaxed on a protein achieving an important function in the cell. Here, we have showed that PGL, sequence evolution rate and CAI are interdependent as already found in eukaryotes [35]. Thus, we believe that the large prokaryotic genomes already available combined with data on gene dispensability, expression, and protein interactivity, open the possibility to new comparative studies testing the prevailing forces driving genome reduction.

### A majority of deletions involving few genes in syntenic fragments

**Figure 6:**
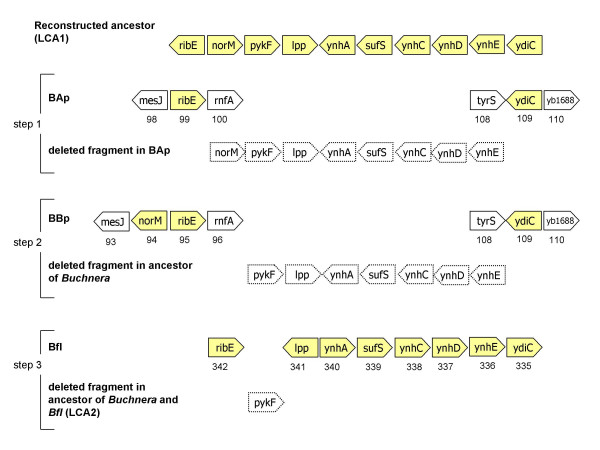
Determination of synteny and deletion events between three extant symbionts (BAp, BBp, Bfl) and their reconstructed free-living ancestor (LCA1), through "alignment" of orthologous genes ordered in each genome. Shaded arrows represent genes from the chosen fragment of LCA1 and still present in symbionts (open symbols for genes in symbionts represent genes from LCA1 present in another fragment) while genes of this fragment that were lost are represented by dotted arrows. The numbers below genes in symbionts show their rank in these genomes. The figure illustrates our approach in three steps: alignment between BAp and LCA1 suggest a non syntenic deletion of 8 genes. Alignment between BBp and LCA1 suggest a non syntenic deletion of 7 genes. Alignment between Bfl and LCA1 finally suggest a syntenic deletion of only 1 gene.

We reconstructed loss events between the last common ancestor of free living and symbiotic bacteria (LCA1) and the last common ancestor of *B. aphidicola *strains (LCA3) or between LCA1 and the supposed common ancestor of symbiotic bacteria (LCA2). Figure 6 gives one example of our approach, where a deletion between LCA1 and *B. aphidicola *BAp is re-analysed after considering gene content of other *B. aphidicola *strains (i.e. in the LCA1 to LCA3 reconstruction of gene losses) or of other symbionts, Bfl and Wgl (i.e. in the LCA1 to LCA2 reconstruction). Using information from the two other *B. aphidicola *strains made little difference given the small number of additional genes they contributed to the reconstruction of LCA3. The common ancestor of the *B. aphidicola *strains possessed only 32 additional genes not found in BAp. Also, in both cases, and as found already by Moran & Mira (2001), a large majority of the losses were in non-syntenic fragments. In sharp contrast, and compared to previous studies [6,7], many fewer genes were lost in the transition from LCA1 to LCA2 because Wgl and Bfl possess 280 genes apparently absent from LCA3. Besides, this step showed a complete inversion of the proportion of genes lost in syntenic vs non-syntenic fragments. This is mainly caused by the fact that gene order was more conserved between *E. coli *and Bfl than in any other comparison as already described by Belda *et al*. (2005) and Degnan *et al*. (2005). Therefore, adding Bfl in the analysis allows the re-establishment of synteny for many fragments (Figure 6).

**Figure 7:**
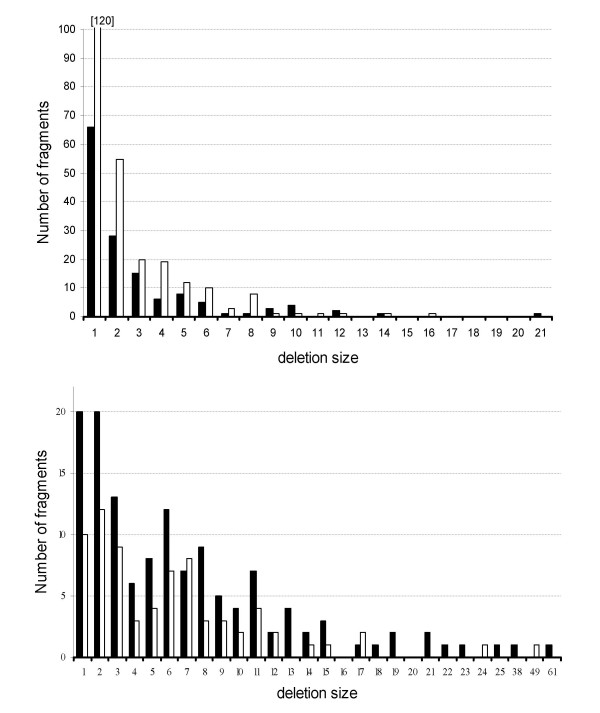
Distribution of deletion sizes in syntenic (upper graph) and non-syntenic (lower graph) fragments between LCA1 and LCA3 (filled bars) or between LCA1 and LCA2 (open bars).

We also compared the size distribution (in number of loci) of losses for the LCA1–LCA3 and LCA1–LCA2 steps for both syntenic and non-syntenic losses (Figure 7). Because a greater fraction of gene losses appeared to be in synteny for the LCA1–LCA2 comparison, most classes of deletion sizes were more represented compared to the LCA1–LCA3 case. However, this was not seen for "large" deletions (10 genes or above) since the LCA1–LCA2 analysis shows only 5 such instances (versus 8). The curves for non-syntenic losses showed an inverse pattern with respect to quantities (fewer non-syntenic losses in the LCA1–LCA2 comparison). But again, the decrease was particularly marked for large deletion size classes: if we actually focus on deletions of 10 genes or above, only 14 remain in the LCA1–LCA2 comparison (36 for LCA1–LCA3). Considering the information from all the symbionts "breaks" many apparently long non-syntenic stretches of genes lost into shorter stretches (N = 340). Reconstructing an hypothetical genome ancestral to different symbionts also led to reduced total number of gene losses, and to smaller blocks of deleted genes. Previous conclusions based on a comparison between a single *B. aphidicola *genome and *E. coli *found on the contrary that early gene loss was supposed to result from large and random deletions [6,11,28]. If the hypothesis of a common origin for these symbioses is accepted, this shows that reductive evolution was from the beginning most often the result of small deletions involving one to five or six gene.

**Figure 8:**
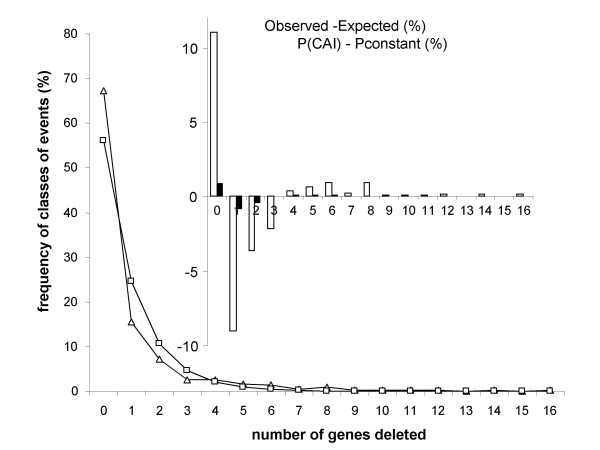
Frequency of classes of deletions sizes (in number of genes) between LCA1 and LCA2, for syntenic fragments. Open triangles, observed frequencies. Open squares, expected frequencies if losses were random and deletions sizes followed a Pascal (geometric) law of mean P equal to the frequency of genes in syntenic fragments actually lost (P = 0.438). Inbedded histogram: i) open bars represent the difference in % between observed frequencies and simulated frequencies of size classes (averages of n = 150 simulations) for a constant probability of loss (H1 hypothesis) ii) open bars represent the difference in % between simulated frequencies for a probability of loss function of the CAI (H2 hypothesis) and for simulated frequencies under H1. The class "zero" corresponds to two adjacent syntenic genes conserved in LCA2 (i.e. no deletion occurred).

To test if deletions were random or clustered, we analysed the distribution of syntenic losses between LCA1 and LCA2 (Figure 8). In the former case, deletion sizes should follow a Pascal (geometric) law of mean P equal to the proportion of genes lost (P = 0.438). In the second case, we should expect long stretches of contiguous genes from LCA1 lost or maintained in the symbionts. We considered the events of two adjacent genes from LCA1 conserved in LCA2 which constitute the class "zero" of deletion sizes, and found that observed and expected curves were significantly different (P < 0.0001). This was due in part to an excess of "large" deletions of 10 genes or above but even more to an excess of the "zero" class. The fact that more regions with contiguous genes were conserved than expected by chance could be explained by the effect of the level of codon bias (CAI) on the probability of loss (as shown above), given that genes with high or low CAI respectively tend to be clustered in the *E. coli *genome (for example ribosomal genes, with high CAI, are organised in clusters). We tested this effect by using a subset of the LCA1 genome, composed of the fragments syntenic with LCA2, and evaluating for that subset the relation between CAI and the probability of loss. We fitted this curve with a sigmoid equation of the form P(CAI)=K1+a e−r(1−CAI)
 MathType@MTEF@5@5@+=feaafiart1ev1aaatCvAUfKttLearuWrP9MDH5MBPbIqV92AaeXatLxBI9gBaebbnrfifHhDYfgasaacH8akY=wiFfYdH8Gipec8Eeeu0xXdbba9frFj0=OqFfea0dXdd9vqai=hGuQ8kuc9pgc9s8qqaq=dirpe0xb9q8qiLsFr0=vr0=vr0dc8meaabaqaciaacaGaaeqabaqabeGadaaakeaacqWGqbaucqGGOaakcqWGdbWqcqWGbbqqcqWGjbqscqGGPaqkcqGH9aqpdaWcaaqaaiabdUealbqaaiabigdaXiabgUcaRiabdggaHjabbccaGiabdwgaLnaaCaaaleqabaGaeyOeI0IaemOCaiNaeiikaGIaeGymaeJaeyOeI0Iaem4qamKaemyqaeKaemysaKKaeiykaKcaaaaaaaa@4373@ and simulated losses in the LCA1 genome that followed a probability function of the CAI of the gene. We averaged n = 150 simulations to evaluate the distribution of deletion sizes obtained in this way and the mean fraction of genes lost (P' = 0.432). We then again simulated losses in the LCA1 genome, assuming a constant probability of loss for each gene, equal to P'. The resulting differences between the two hypotheses, i.e. a probability of loss being either a function of CAI or being constant across the genomes actually show that considering the effect of CAI and the clustering of genes with similar CAI does result in a slight excess of regions without deletions and of regions with "large" deletions (more than 5 genes, Figure 8, imbedded diagram). However, these differences are small when compared to the differences between observed and expected distributions under a random expectation (Figure 8), so the clustering of genes with a similar CAI only explains a small part of the clustering of deletions. Other reasons may in fact explain that genes were not lost in a strictly random way throughout the genome: genes are often in operons, the whole operon being either lost or conserved altogether. This result on clustering of deletions is consistent with previous findings [6]; however, in our case, discrepancies between the observed distribution of deletions and a "neutral" model of deletions were mostly seen for blocks of 2–5 genes. These conclusions concern losses in syntenic fragments (although these could now represent the majority of losses), and it may still be imagined that a few large blocks were lost in non-syntenic fragments, through processes mostly governed by drift. Finally, our result are fully consistent with the "domino" theory of gene extinction in bacterial genomes [42], which posits than in a first step all genes are under an even threat to be knocked out by mutations, which results in a second step by mass decay of depending genes involved in the same pathway. This would also explain the physical clustering of deletions involving a few genes as found in our study, while no or few large blocks would be lost.

### Validity of the results under alternative phylogenetic scenarios

We stress that even under the hypothesis of independent origins of endosymbionts, our conclusions on the links between gene dispensability and selective parameters (CAI, Ka) would be little affected. Indeed, a strong effect of CAI and Ka on the propensity of gene loss was observed even without considering the existence of LCA2 because it was observed for the losses occurring between LCA1 and each of the extant endosymbiotic lineages.

In contrast the reconstruction of deletion sizes is more dependent on the validity of our common ancestry of the five symbionts studied. Considering the reconstructed ancestor of the three *B. aphidicola *strains that are clearly monophyletic affected only marginally earlier studies based on single genome [6].

## Conclusion

The particular originality of this work compared to that of precedent studies [6,7] lies in its integration of the information on the selective pressures on genes together with more genome data. The larger genome data set allowed us to detect and characterize more ancient events of gene loss by including the reconstructed common ancestors of symbiotic lineages. We have shown that genes lost in the early stages of symbiosis are on average less selectively constrained than genes conserved in any of the extant symbiotic strains studied. This is shown by significant differences among the two types of genes of two parameters that can measure selective importance: non-synonymous evolutionary rates (in symbionts or in free-living enterics) and codon bias in *E. coli*. In addition, our reconstruction of deeper nodes allowed also a better description of deletion events at the different steps, in particular of their size distribution. Under the hypothesis of a common origin of different symbioses, gene losses would have been mostly occurring through rather small blocks, and in syntenic regions between at least one of the symbionts and present-day *E. coli*. Our study did not include two genomes from insect-associated endosymbionts that have just been completed, *Blochmannia pennsylvanicus *[3] and *B. aphidicola BCc *(host C*inara cedri*, available soon). Studying these genomes will help to better reconstruct recent gene loss events, which occurred after the divergence of *Blochmannia *and *B. aphidcola *strains respectively. However, given their small genomes that include very few genes not already present in other complete sequences, their inclusion could not significantly change our conclusions on early patterns of gene loss.

The knowledge of more endosymbiotic genomes, particular of genomes of larger sizes (e.g. *Sitophilus oryzae *primary endosymbiont [23] will be of paramount interest for fully resolving several puzzles that remain to date. It will indeed provide a more robust phylogenetic scenario of symbiosis acquisition (in single or multiple events) and a finer knowledge on the rate and patterns of gene losses, which will allow disentangling mutational and selective pressures that modulate genome reduction.

## Methods

### Genomic sequences

Complete genomes were retrieved from EMBL database: *Escherichia coli *K12 (NC_000913), *Salmonella typhimurium *LT2 (NC_003197), *Vibrio cholerae *O1 biovar eltor str. N16961 (NC_002505, NC_002506), *Yersinia pestis *(NC_003143), *Buchnera aphidicola *of *Baizongia pistaciae *(NC_004545), *Buchnera aphidicola *of *Schizaphis graminum *(NC_004061), *Buchnera *aphidicola of *Acyrthosiphon pisum *(NC_002528), *Wigglesworthia brevipalpis *(NC_004344), *Candidatus Blochmannia floridanus *(NC_005061). The symbiotic strains will be referred as *B. aphidicola *BAp, *B. aphidicola *BSg, *B. aphidicola *BBp, *Wigglesworthia *(Wgl) and *Blochmannia *(Bfl).

### Phylogenetic tree

Phylogenetic reconstruction of trees including endosymbiotic DNA sequences which are strongly AT-biased and evolve at relatively high rates is problematic when using classical models of DNA sequence evolution. To avoid this pitfall, we used the tree building method implemented in NHML3 which is based on a heterogeneous model accounting for unequal transition/transversion rates, unequal evolutionary rates among sequence sites and unequal base compositions of sequences [43]. Maximum likelihood inference based on this model was applied to a trimmed alignment of 61 concatenated conserved protein-coding genes (19143 nucleotides) involved in translation. Trimming was done with GBLOCKS [44] in order to limit the data set to unambiguous well conserved parts of the alignments. Only the first two positions of codons, which are relatively less AT enriched [15] were retained for phylogenetic analysis. This yielded a most likely phylogenetic tree that grouped together the five endosymbiotic lineages (Figure 1) suggesting a unique origin of endosymbionts. As the calculation time of this phylogenetic method impeded bootstrap analysis, we decided to test alternative tree topologies, for example assuming several acquisitions of endosymbiosis. We also tested slightly different topologies resulting from the move of *Yersinia pestis *and *Salmonella thyphimurium *along the tree. Loglikelihood differences between all additional trees and the initial topology were tested using the loglikelihood ratio tests.

### Reconstruction of endosymbiotic ancestors

Because it is difficult to assign orthology for structural RNA, we excluded from the analysis all non coding genes. To determine the set of orthologous coding genes between *E. coli *K12 and each of the nine bacteria, we performed reciprocal blasts with a cut off value of 10^-4^, retaining only those genes that were best hits in both comparisons. Applying a parsimony principle, the common ancestor of all endosymbionts was reconstructed as the sum of the orthologous coding genes present in at least one endosymbiotic lineage, *E. coli *and *V. cholera*. This led to removal of the few genes that were present in endosymbionts but have no orthologous equivalent in either *E. coli *or *V. cholera*. Pseudogenes of endosymbiotic genomes were included in the analysis and considered as lost genes.

Finally, this approach allowed the determination of a minimal free-living ancestor of endosymbionts and of their free-living relatives (LCA1) comprising 1983 conserved coding genes that have a low probability of being acquired by lateral gene transfer (LGT). Indeed, LGT generally involves uptake, from distantly related bacteria or phages [45], of genes that are absent from related bacteria. Since our method for reconstructing LCA1 was very conservative, we can speculate that the real last free-living bacteria that gave rise to endosymbionts contained more genes. Intermediary common ancestor to symbiotic lineages were defined as subsets of the initial 1983 CDS of LCA1: LCA2 corresponded to the sum of genes present in all endosymbiotic lineages, generating our common ancestry to all these symbionts, LCA3 to the sum of the genes present in all *B. aphidicola *lineages, and LCA4 to the sum of genes present in *Blochmannia *and *Wigglesworthia *lineages (Figure 1).

### Quantifying gene loss

Gene loss was quantified using different approaches including both quantitative and qualitative variables.

*A quantitative marker of gene loss*: for each gene present in LCA1, a parameter called the propensity of a given gene to be lost (PGL) was calculated [35]. This measure required the identification of gene loss along the branches of the phylogeny which was based on the reconstruction of last common ancestors of endosymbionts described above. The propensity of a gene to be lost was calculated as the ratio of the sum of the lengths of branches lacking a given gene to the sum of the lengths of all branches of the tree. This generated a quantitative parameter ranging from 0 (never lost) to 1 (lost from all the branches) that could be used to perform correlations with CAI and substitution rates (Tables 1 and 2).

*Discrete categories of genes *(Figure 3): we quantified gene loss by grouping genes present in LCA1 into three categories: (A) genes lost in all symbionts, presumably between LCA1 and LCA2; (B) genes present in LCA2 but lost in some of the symbiotic lineages; (C) genes kept in all endosymbionts.

### CAI and substitution rate estimates

Our main objective was to examine correlations between patterns of gene loss (at different depths of the tree) and the functional importance of genes, in order to measure if losses, particularly in the initial stages of symbiosis could have been limited by rather precise constraints [7]. To evaluate the level of functional importance of genes, we used two different parameters i) the level of adaptive codon bias (CAI) and ii) the rate of sequence evolution. The former parameter is correlated with the level of gene expression in *E. coli *[46], and more essential genes probably have higher levels of expression [47]. Unfortunately CAI data are not available for genes of endosymbionts which show hardly any trace of adaptive bias [12,15]. We therefore used the CAI of *E. coli *orthologs, calculated through the CODONW package [48].

In addition, we estimated synonymous (Ks) and non-synonymous (Ka) substitutions rates by performing pairwise comparisons of coding sequences. Estimates were calculated using Li's method [49] implemented in the diverge function from the GCG 10.2 package. Since Ks were likely saturated for many of the pairwise comparisons, we restricted our analysis to non-synonymous substitution rates (Ka). Pairwise estimates of non-synonymous substitution rates were conducted for two free-living species pairs (Eco-Stm, Eco-Ype) and four endosymbiotic species pairs (BAp-BSg, BAp-BBp, BSg-BBp, Bfl-Wgl; see Schaber *et al*. [40] for detailed results on substitution rates).

Finally, we looked at the relation between gene loss at different depths of the tree and the degree of functional constraint of genes (estimated by CAI or evolutionary rates) (Figures 3, 4, 5). The significance of the different relationships was tested using either the non-parametric Mann-Whitney tests or the Spearman correlation tests.

### Reconstruction of gene deletions

Following Moran and Mira [6], we assumed that the LCA1 possessed the *E. coli *gene order. This is likely true for a majority of genes, given evidence discussed by these authors that most gene rearrangements occurred in the symbiont, and is also supported by our estimations that about 75% of the genes from chromosome I in *V. cholerae *are in syntenic fragments with *E. coli*. Synteny was defined between fragments of the LCA1 and individual symbiotic species if consecutive genes in the LCA1 corresponded to consecutive genes in a given symbiont. To reconstruct synteny between LCA1 and the ancestor of *B. aphidicola *strains (LCA3), we used the fact that gene order is conserved in that group and simply "filled the gaps" of *B. aphidicola *BAp with genes absent from this species but present in either of the two other *B. aphidicola *strains. It was not possible to establish the gene order in LCA2 given the major rearrangements between *Blochmannia*, *Wigglesworthia*, and *Buchnera *(LCA3). However, we assumed that if synteny was established between a fragment in LCA1 and genes from any of these three symbionts, this fragment must have been syntenic with LCA1 in the common ancestor of these symbionts (LCA2).

## Authors' contributions

FD, CR and JS conceived of the study and carried out the statistical analysis. FJS participated in the design of the study. FD drafted the manuscript. AM initiated the study and participated in its coordination. All authors read and approved the final manuscript.
